# Research progress of fibroblast growth factor 23 in acute kidney injury

**DOI:** 10.1007/s00467-022-05791-z

**Published:** 2022-11-22

**Authors:** Lina Zhang, Wei Qin

**Affiliations:** 1 Division of Nephrology, Department of Medicine, West China Hospital, Sichuan University, 37 Guoxue Lane, Chengdu, 610041 Sichuan China; 2 Division of Nephrology, Henan Key Laboratory for Kidney Disease and Immunology, Henan Provincial People’s Hospital, Zhengzhou, Henan China

**Keywords:** Fibroblast growth factor 23, Acute kidney injury, Biomarker, Treatment

## Abstract

Fibroblast growth factor 23 (FGF23) is primarily produced in bones and mainly regulates calcium and phosphorus metabolism. The level of circulating FGF23 increases rapidly in the early stage of acute kidney injury (AKI). Recent studies have shown that FGF23 may serve as a biomarker for the diagnosis and poor prognosis of AKI. The mechanism of increased FGF23 in AKI may include increased production of FGF23, decreased renal clearance of FGF23, and some new regulatory factors, such as inflammation and glycerol 3-phosphate. However, the biological effects of elevated FGF23 in AKI are still unclear. It is also not known whether reducing the level of circulating FGF23 could alleviate AKI or its poor prognosis. Here, we review the pathophysiological mechanism and possible regulation of FGF23 in AKI and discuss the possibility of using FGF23 as a therapeutic target.

## Introduction

Acute kidney injury (AKI) is a syndrome with a sharp decline of glomerular filtration. Epidemiological surveys have reported that the prevalence of AKI ranges from < 1 to 66% [1]. The prognosis of AKI is not optimistic as it can lead to chronic kidney disease (CKD). The mortality of hospitalized patients with AKI including adults and children ranges from 8.8 to 12.4% [2–4], while the mortality of AKI patients in the intensive care unit (ICU) during hospitalization is even higher [5], and can be as high as 37% [6]. Because of the high incidence and mortality of AKI mentioned above, early identification of AKI and timely intervention are particularly important. Early diagnosis and intervention of AKI can provide better treatment options, improve clinical outcomes, and reduce mortality. Therefore, researchers need to search for rapid and sensitive biomarkers for the early diagnosis and treatment of AKI.

Fibroblast growth factor 23 (FGF23) was originally discovered as a vital regulator of phosphate and calcium metabolism. It is significantly increased in patients with CKD, and it has become an important biomarker of cardiovascular disease [7, 8]. In recent years, it has been reported that circulating FGF23 levels are also significantly elevated in patients with AKI [9, 10]. This suggests that FGF23 may become a novel early sensitive biomarker of AKI. Here, we review the latest research progress related to FGF23 in AKI, including the potential mechanisms for the increase, and discuss the possibility of targeting FGF23 in AKI.

## Physiology of FGF23

### Basic information of FGF23

**Fig. 1:**
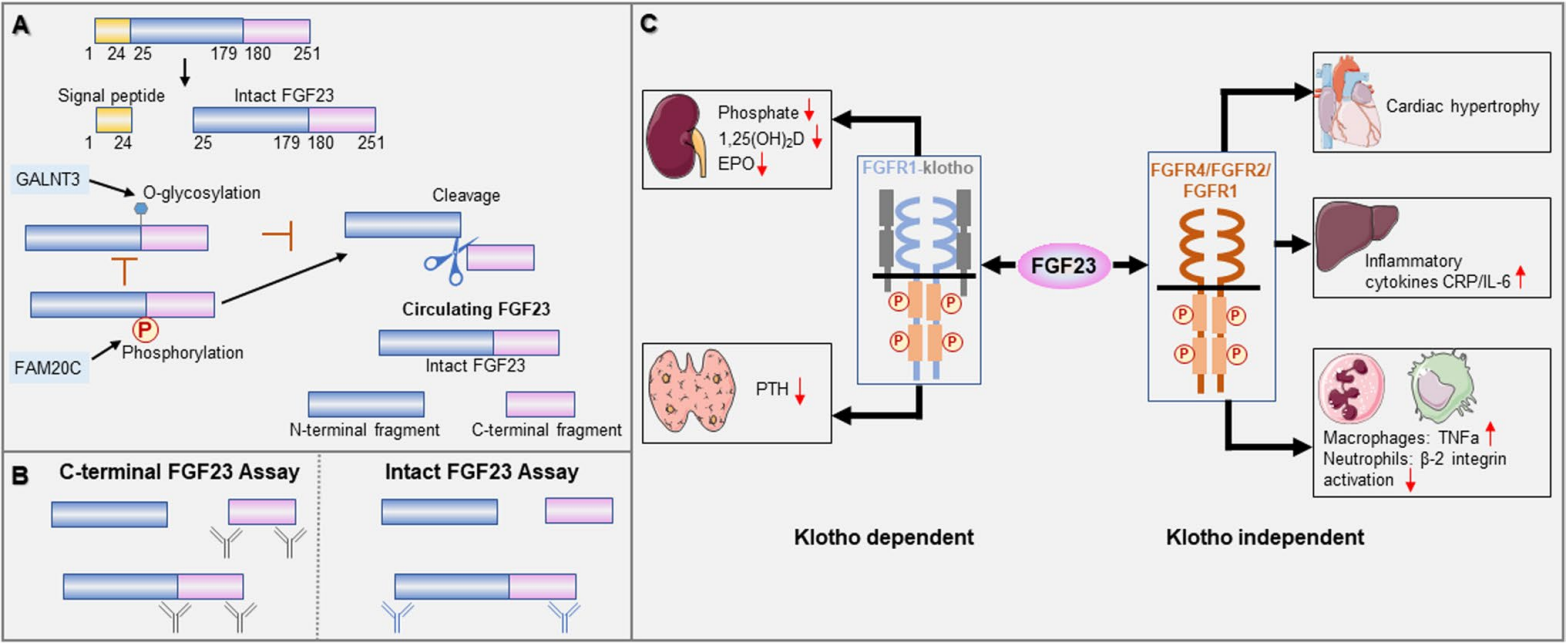
Structure, serological assays, functions of FGF23 and its pathway.** A** FGF23 is a 32 kDa glycoprotein that contains 251 amino acids. After a 24-amino acid signal peptide is cleaved, the full-length protein with 227-amino acid is excreted into the circulation. FGF23 can be proteolytically cleaved between Arg179 and Ser180 to generate N-terminal and C-terminal fragments. O-glycosylation by N-acetyl-galactosaminyl transferase 3 (GALNT3) at Thr178 stabilizes FGF23. Phosphorylation by the extracellular serine/threonine protein kinase FAM20C at Ser180 prevents O-glycosylation by GALNT3 and induces proteolytic cleavage of FGF23. **B** Circulating FGF23 levels can be measured by either intact FGF23 assay or C-terminal assay. Intact FGF23 assays use antibodies that recognize two epitopes flanking the proteolytic cleavage site and therefore exclusively capture the intact molecule. C-terminal FGF23 assays detect both intact FGF23 and its carboxy-terminal fragments by recognizing two epitopes in the carboxyl terminus distal to the cleavage site. **C** Functions of FGF23 in different tissues and its pathway. FGF23 reduces serum phosphate, 1,25(OH)_2_D, EPO, and PTH in klotho-dependent manner. In addition, FGF23 induce cardiac hypertrophy, stimulate the secretion of inflammatory cytokines such as CRP and IL-6, inhibit neutrophil β-2 integrin activation, and induce the expression of TNFα in macrophage in klotho-independent manner. 1,25(OH)_2_D, 1,25-dihydroxyvitamin D; EPO, erythropoietin; PTH, parathyroid hormone; CRP, C-reactive protein, IL-6, interleukin-6; TNFα, tumor necrosis factor α; ↓, decrease; ↑, increase

FGF23 is mainly produced in bone, including osteocytes, osteoblasts, and bone marrow [11, 12]. However, it has recently been reported that in the case of heart and kidney disease, FGF23 can also be secreted by other organs and cells, such as cardiomyocytes, renal tubular epithelial cells, spleen, or vascular endothelial cells [13–16]. Human FGF23 is a 32 kDa glycoprotein that contains 251 amino acids. After a 24-amino acid signal peptide is cleaved, the 227-amino acid FGF23 protein is excreted into the circulation (Fig. 1A). This full-length protein is thought to be a bioactive hormone.

### Posttranslational modification of FGF23

Posttranslational modifications may affect circulating FGF23 levels. O-glycosylation by N-acetyl-galactosaminyl transferase 3 (GALNT3) at Thr178 stabilizes FGF23. In contrast, phosphorylation by the extracellular serine/threonine protein kinase FAM20C at serine (Ser) 180 prevents GALNT3-mediated O-glycosylation and induces proteolytic cleavage of FGF23 (Fig. 1A). Therefore, the balance between posttranslational glycosylation and phosphorylation is an important factor affecting the proportion of full-length FGF23 and its fragments in circulation [17, 18].

FGF23 that is not modified by O-glycosylation can be cleaved between arginine (Arg) 179 and serine (Ser) 180 to generate an N-terminal fragment and a C-terminal fragment [19, 20]. Thus, a variety of FGF23 peptides are present in the blood, including full-length FGF23 and N-terminal and C-terminal fragments.

### Measurement of FGF23

The concentration of circulating FGF23 is mainly determined by ELISA. At present, there are two kinds of kits available, one is intact FGF23 (iFGF23) detection kit and the other is C-terminal FGF23 (cFGF23) detection kit [21, 22]. These assays are very different from each other, using antibodies targeted to different epitopes of the FGF23 protein. The iFGF23 assays use two antibodies, one recognizes the N-terminal domain epitope and the other binds to the C-terminal domain and detects the intact protein. For cFGF23 detection, intact FGF23 and C-terminal fragments are captured since two antibodies bind to two different epitopes in the C-terminal region (Fig. 1B).

Moreover, these two kinds of kits use different units of measurement: iFGF23 in picograms per milliliter (pg/ml) and cFGF23 in relative units (RU) per milliliter (RU/ml). Due to the different calibrators used in the kits from different manufacturers, even the absolute values of the same detection method vary widely [23, 24]. In addition, reference ranges for FGF23 detecting results are not well defined yet [22, 25, 26]. These unsolved problems limit the routine clinical application of FGF23 test kits.

### Functions of FGF23 and its pathway

Previous studies have widely confirmed that the main function of FGF23 is to regulate calcium and phosphorus metabolism. FGF23 could inhibit proximal tubular phosphate reabsorption by inhibiting sodium phosphate transporters, reduce serum 1,25(OH)_2_D by inhibiting cytochrome Cyp27b1 to reduce its production and stimulating Cyp24A1 to increase its degradation [27, 28]. In addition, FGF23 could also suppress the secretion of parathyroid hormone (PTH) [29]. These physiological roles of FGF23 are mediated by binding to its receptor FGFR and its co-receptor klotho [30].

However, recent research has shown that FGF23 has multiple distinct effects on other tissues, sometimes in a klotho-independent manner, particularly under pathological conditions such as CKD. In cardiomyocytes without klotho expression, FGF23 acts on FGFR4 and subsequently triggers PLCγ/calcineurin/NFAT signaling pathway to induce cardiac hypertrophy [31]. In the liver, FGF23 increases production of inflammatory cytokines such as C-reactive protein (CRP) and interleukin-6 (IL-6) by activating the FGFR4/PLCγ/calcineurin/NFAT pathway, independent of klotho [32]. In the kidney, FGF23 acts on FGFR/klotho complex to suppress the expression of hypoxia-inducible factor-1α (HIF-1α), thereby reducing renal erythropoietin (EPO) secretion in patients with CKD [33, 34]. In addition, it has been reported that FGF23 could impair immune responses and host defense in CKD patients. The evidence was that FGF23 could act on FGFR2 to inactivate β2-integrin function then inhibit neutrophil recruitment [35], and promote the expression of tumor necrosis factor-α (TNF-α) in macrophages [36]. The effect of FGF23 in multiple organs and its pathways are shown in Fig. 1C.

## FGF23 and AKI

### Alterations of FGF23 in AKI

Elevated circulating FGF23 in AKI was first reported in a patient with rhabdomyolysis [37]. Subsequently, Leaf et al. [38] conducted a case–control study and found that plasma cFGF23 levels were significantly higher in patients with AKI compared with those without AKI (1471 [224–2534] vs. 263 [96–574] RU/mL). In the following years, a series of clinical studies have explored the alterations of FGF23 in AKI of different etiologies. In most studies, researchers have reached the conclusion that FGF23 levels were significantly elevated in AKI patients. We summarize the clinical findings to date in Table 1.

**Table 1:** Clinical studies of FGF23 and AKI

Study	Subjects	Age	AKI definition	Sample	FGF23 assay	Time of FGF23 assay	Predict AKI	Predict adverse outcome
Leaf et al., 2012 [38]	30 AKI and 30 controls from ICU	Adults	AKIN	Plasma	cFGF23	cFGF23 measured within 24 h of AKI onset	N/A	OR* for KRT/mortality 13.73 (1.75–107.5)
Ali et al., 2013 [61]	19 children underwent CPB	Children	pRIFLE	Plasma	cFGF23	Preoperative cFGF23	> 86 RU/ml were associated with a twofold higher risk of AKI	N/A
Brown et al., 2014 [62]	3241 patients in community aged ≥ 65 years	Adults	ICD-9-CM	Plasma	cFGF23	cFGF23 measured at enrollment	HR* 1.99 (1.04–3.80)	N/A
Leaf et al., 2016 [39]	250 patients undergoing cardiac surgery	Adults	KDIGO (SCr only)	Plasma	iFGF23 cFGF23	cFGF23 measured at the end of CPB	OR 1.42 (1.03–1.95)	OR* for KRT/death 1.65 (0.99–2.75); vasopressors 2.94 (2.02–4.27); sepsis 1.60 (1.13–2.28)
Leaf et al., 2017 [63]	350 ICU patients	Adults	KDIGO	Urine	cFGF23	urinary cFGF23 measured within 24 h of admission	OR for AKI/death 3.9 (1.6–9.5)	OR* for hospital mortality 1.46 (1.09–1.97) 90-day mortality 1.31 (1.00–1.72) 1-year mortality 1.44 (1.10–1.90)
Bai et al., 2018 [43]	144 children in ICU	Children	AKIN	Serum Urine	iFGF23	N/A	FGF23 levels could not predict AKI in critically ill children	N/A
Leaf et al., 2018 [44]	(1) 817 patients with AKI requiring KRT (ATN study) (2) 710 patients with and without AKI (VALID study)	Adults	KDIGO	Plasma	iFGF23 cFGF23	FGF23 measured on enrollment	N/A	OR* for death (1)ATN study cFGF23 3.84 (2.31–6.41) iFGF23 2.08 (1.03–4.21) (2)VALID study cFGF23 3.52 (1.96–6.33) iFGF23 1.93 (1.12–3.33)
Rygasiewicz et al., 2018 [41]	79 ICU patients	Adults	KDIGO	Plasma	iFGF23 cFGF23	cFGF23 measured within 24 h of ICU admission	cFGF23 > 135.91 RU/mL predict AKI, OR 1.80 (1.10–2.96)	OR* for hospital mortality 2.85 (1.60–5.06)
Sakan et al., 2018 [64]	121 AKI patients after major surgery	Adults	KDIGO	Plasma	cFGF23	cFGF23 measured on the first postoperative day	> 285 RU/mL on POD1 could predict AKI	> 709 RU/mL predict overall mortality in 6-months
Shaker et al., 2018 [42]	80 patients underwent cardiac surgery	Adults	AKIN	Plasma	iFGF23	Percent change in iFGF23 measured before and 24 h after surgery	Percent change of FGF23 > 435.24% predicts AKI (AUC 0.9, sensitivity 100%, and specificity 97.1%)	N/A
Volovelsky et al., 2018 [65]	41 infants underwent CPB	Children	pRIFLE	Serum	cFGF23	cFGF23 assessed 4–8 h after CPB	Serum FGF23 could predict AKI, with AUC 0.74 (0.50–0.90)	N/A
Volovelsky et al., 2018 [10]	83 children with congenital heart disease	Children	KDIGO	Serum	cFGF23	Preoperative cFGF23 measured within 3 days of surgery	OR 7.5 (1.03–79.3)	N/A
Wu et al., 2018 [66]	257 patients with AKI undergoing KRT after CPB	Adults	Scr > 50% baseline during the first three PODs	Plasma Urine	iFGF23 cFGF23	plasma cFGF23 measured at start of dialysis	N/A	> 2050 RU/mL independently associated with higher 90-day mortality (HR 1.76)
de Oliveira Neves et al., 2019 [67]	265 ICU patients	Adults	KDIGO	Plasma	cFGF23	cFGF23 measured within 24 h of ICU admission	OR 1.70 (1.19–2.40)	N/A
Hanudel et al., 2019 [46]	161 pediatric ARDS patients	Children	pRIFLE	Plasma	iFGF23 cFGF23	cFGF23 measured within 24 h of ARDS diagnosis	OR 1.52 (1.02–2.26)	OR* for 60-day mortality 1.62 (1.07–2.45)
Neyra et al., 2019 [68]	54 AKI patients and 52 matched controls from ICU	Adults	KDIGO	Serum	iFGF23	iFGF23 measured 24–48 h after AKI diagnosis	N/A	OR* for 90-day outcome (death, KRT, 50% decrease in eGFR) 4.48 (1.29–15.53)
Chang et al., 2020 [45]	149 CKD patients undergoing KRT for AKI	Adults	KDIGO	Plasma Urine	cFGF23 iFGF23	cFGF23 measured just before dialysis	N/A	HR* for 90-day mortality 2.5 (1.5–4.1)
Pramong et al., 2020 [9]	62 patients with ADHF	Adults	KDIGO	Plasma	cFGF23	cFGF23 measured at 24 h after ADHF diagnosis	> 450 RU/mL predicts AKI (sensitivity 71.4%, specificity 61.8%)	N/A

*FGF23* fibroblast growth factor 23, *AKI* acute kidney injury, *ICU* intensive care unit, *AKIN* acute kidney injury network, *cFGF23* C-terminal FGF23, *N/A* no available information, *OR* odds ratio, *KRT* kidney replacement therapy, *CPB* cardiopulmonary bypass, *pRIFLE* pediatric modified RIFLE (Risk, Injury, and Failure; and Loss; and End-stage kidney disease), *ICD-9-CM* International Classification of Disease, Ninth Revision, clinical modification diagnosis codes, *HR* hazard ratio, *KDIGO* Kidney Disease: Improving Global Outcomes, *Scr* serum creatinine, *iFGF23* intact FGF23, *AUC* area under the curve, *POD* postoperative day, *ARDS* acute respiratory distress syndrome, *eGFR* estimated glomerular filtration rate, *ADHF* acute decompensated heart failure

^*^OR or HR values after adjustment

As mentioned above, two kinds of ELISA kits are mainly used to detect circulating FGF23 levels: the cFGF23 assay kit captures both intact FGF23 and C-terminal fragments, while the iFGF23 assay captures only the intact FGF23. Leaf et al. [39] measured cFGF23 and iFGF23 and found that while both cFGF23 and iFGF23 were elevated in AKI, the level of cFGF23 was almost 25–75 times higher, while iFGF23 was only two times higher in AKI patients than in non-AKI patients. A cohort study of 32 pediatric cardiac surgery patients reported similar findings [40]. These data show that cFGF23 levels were significantly increased and out of proportion to iFGF23 levels. This may be due to declined clearance of FGF23 fragments in AKI or reduced processing of FGF23 by damaged kidneys in AKI. Another reason may be that both FGF23 production and cleavage are significantly increased in AKI.

Notably, Leaf et al. [39] reported that cFGF23 levels increased in both patients with and without AKI, although the increase was higher in patients with AKI. Circulating cFGF23 was found to be elevated even in patients without AKI, suggesting that these patients may have experienced subclinical renal injury or that cFGF23 may be a product of post-operative stress. Hanudel et al. [40] assessed the oxygen saturation and found that chronic hypoxemia was associated with elevated FGF23 levels, suggesting that increased circulating cFGF23 levels may be one of the markers for hypoxemia.

### Diagnostic value of FGF23 in AKI

Increased circulating FGF23 was reported to be a diagnostic marker of AKI. Rygasiewicz et al. [41] conducted a cohort study of 79 ICU patients and revealed that cFGF23 concentrations above 136 RU/mL measured within 24 h of ICU admission were able to predict AKI with an area under the curve (AUC) of 0.81 (sensitivity 83% and specificity 82%). Shaker et al. [42] detected plasma iFGF23 in 80 adult patients who underwent cardiac surgery and reported that percent change of FGF23 > 435% measured before and 24 h after surgery could predict the occurrence of AKI (AUC 0.9, sensitivity 100%, and specificity 97.1%). Other studies have confirmed the predictive value of FGF23 in AKI diagnosis (Table 1). However, in a prospective cohort study in 144 pediatric ICU patients, Bai et al. [43] found that blood and urine FGF23 levels did not significantly correlate with AKI occurrence. This does not agree with the findings of previous studies. In their study, Bai et al. recruited children admitted to pediatric ICU, regardless of diagnosis, aged between 1 month and 16 years. In this study, FGF23 levels inversely correlated with age in children younger than three years old, but not in older children. Thus, analysis of the correlation between FGF23 and AKI in children including both under and over three years of age cannot avoid the influence of age on FGF23. Perhaps further subgroup analysis by age group may yield meaningful results about the correlation between serum FGF23 and AKI.

To date, clinical studies have shown that circulating FGF23 levels, especially cFGF23 levels, are significantly elevated in patients with AKI. Increased serum FGF23 has been demonstrated to be a predictor of AKI. However, the diagnostic predictive value of FGF23 in AKI remains somewhat controversial, especially for iFGF23. More large cohort studies are needed to explore the role of FGF23 in predicting AKI.

### Value of FGF23 in the prognosis prediction of patients with AKI

In addition to being an early biomarker of AKI, FGF23 may also be a predictor of poor prognosis in patients with established AKI. Leaf et al. [44] measured plasma FGF23 in 1527 patients and found that higher circulating FGF23 levels were associated with higher 60-day mortality. This association existed for both cFGF23 and iFGF23 and was independent of age, sex, and other known risk factors. Chang et al. [45] evaluated the relationship between FGF23 and adverse outcomes in 149 patients with CKD superimposed with AKI requiring kidney replacement therapy (KRT); their results showed that higher plasma cFGF23 levels were associated with a high risk for 90-day mortality [hazard ratio (HR) 2.5; *P* < 0.001] even after adjustment for sex, age, baseline eGFR, and disease severity. Another study assessed AKI and clinical outcomes in a multicenter cohort of pediatric patients with acute respiratory distress syndrome; plasma iFGF23 and cFGF23 concentrations were measured, but only cFGF23 levels were associated with 60-day mortality [odds ratio (OR) 1.62, *P* = 0.023] [46], independent of age, sex, and other risk factors. These findings suggest that FGF23 is associated with adverse outcome of AKI in patients in different etiologies.

Although several clinical studies suggest that FGF23 may be a direct toxic factor of AKI, it is still unclear whether FGF23 is merely a biomarker or is directly involved in the pathogenesis of AKI, as there is no experimental evidence. Chang et al. [47] found that FGF23 protein preconditioning in ischemia–reperfusion (IR)-AKI mice improved kidney injury by promoting tubular regeneration, proliferation, and vascular repair, and reducing tubular injury. Further experiments revealed that FGF23 inhibited endothelial progenitor cell senescence and migration in a klotho-independent manner, but did not inhibit angiogenesis. This study proposed a protective effect of FGF23 in the IR-AKI model. Another group reported that the FGF23–klotho signaling pathway could promote the proliferation of renal proximal tubule epithelial cells [48] and inhibit vitamin D-induced apoptosis. These results provide evidence that FGF23 may be a protective factor in kidneys.

Previous observations support the idea that FGF23 may be a promising biomarker of poor prognosis in AKI patients. However, FGF23 may also be an early protective factor against kidney injury. There are still many unanswered questions about the causal relationship between FGF23 and AKI and the underlying mechanisms. We need a better understanding of the physiological and pathophysiological effects and mechanisms of FGF23 in AKI.

### Possible mechanisms of FGF23 elevation in AKI

In a folic acid (FA)-induced mouse model [49], researchers found elevated plasma FGF23 levels as early as 1 h after folic acid injection. Both plasma iFGF23 and cFGF23 levels were elevated in this FA-induced AKI animal model. Increased circulating FGF23 levels have subsequently been reported in other animal models, including those of hemorrhagic shock or sepsis [50], nephrectomy [51], and obstructive nephropathy [14]. However, the causes of the rapid increase in circulating FGF23 should be clarified.

Under normal physiological conditions, FGF23 is mainly secreted in bone. Researchers first evaluated the expression level of FGF23 in bone tissue of FA-induced AKI mice and found that both FGF23 mRNA and protein expression levels were increased [49, 52]. Accordingly, increased bone production may be one of the causes of increased serum FGF23 levels. However, studies also reported ectopic production of FGF23 in other tissues, even at higher levels than bone FGF23 production. In an FA-induced AKI mouse model, Egli-Spichtig et al. [16] found that FGF23 mRNA expression was upregulated 5–15 times in the thymus, spleen, and heart, but only 2 times in bone. Also, the ectopic expression of FGF23 mRNA in kidneys was demonstrated in that study. Mace et al. [14] established a unilateral ureteral obstruction model; while FGF23 mRNA expression was undetectable in normal kidneys, it was already detectable 2 h after the obstruction, with further significant elevations at 4 and 6 h. Subsequent studies have confirmed ectopic production of FGF23 in kidneys, liver, and bone marrow in different animal models of AKI [50, 53]. Taken together, the early rise in circulating FGF23 in AKI is accompanied by upregulated FGF23 expression in multiple organs.

Impaired renal FGF23 clearance may be one of the reasons for the increased levels of FGF23 in AKI. Christov et al. [49] injected recombinant human FGF23 into AKI mice and found that the half-life of FGF23 was prolonged by 50%. Mace et al. [51] performed bilateral nephrectomy in rats and found an immediate increase in plasma iFGF23, with a significant 2–3-fold elevation within only 15 min after nephrectomy. Further experiments demonstrated that the half-life of exogenous recombinant human FGF23 was significantly extended from 4.4 to 11.8 min. Plasma FGF23 measurements of renal arteries and veins have shown that the renal extraction rate of circulating FGF23 is about 40%. These results indicate that impaired renal clearance of circulating FGF23 may lead to elevated circulating FGF23 levels.

The increase of FGF23 was independent of its classical regulators, given that even in mice with PTH or vitamin D receptor depletion, AKI induced by FA caused the elevation of FGF23 [49]. In addition, a low-phosphate diet could not prevent the rise of FGF23 in this AKI model. Similarly, Mace et al. [51] conducted parathyroidectomy before bilateral nephrectomy and found that the increase in FGF23 was not affected. These results have shown that rather than classic regulators such as PTH, vitamin D, and phosphate, additional mechanisms play a role in the elevation of circulating FGF23 in AKI.

It has recently been demonstrated that inflammation may affect FGF23 transcription and cleavage [54]. Injection of IL-6/soluble IL-6 receptor fusion protein hyper IL-6 plasmid elevates circulating FGF23 [55]. It also increases the expression of FGF23 mRNA in cultured calvaria organs and osteoblast-like UMR106 cells. Further experiments have shown that IL-6 enhances FGF23 promoter activity through signal transducer and activators of transcription 3 (STAT3) phosphorylation, and then induces the transcription of FGF23. Radhakrishnan et al. [53] revealed that estrogen-associated receptor γ (ERR-γ) is a novel transcriptional regulator that regulates hepatic FGF23 production in AKI. They investigated FA-induced AKI in mice and found that upregulated systemic IL-6 induced the expression of hepatic ERR-γ. Furthermore, hepatocyte-specific genetic depletion of ERR-γ or ERR-γ inverse agonist reduced hepatic and circulating FGF23 levels in AKI mice. In other words, AKI induces an increase in plasma IL-6 levels, which in turn increases liver ERR-γ and FGF23 gene expression, thereby promoting an increase in plasma FGF23 levels.

There have been some other new directions regarding the possible mechanisms of FGF23 elevation in AKI. A recently published study has suggested that a protein released by kidneys may stimulate the production of FGF23 in AKI [56]. Glycerol-3-phosphate (G-3-P), a byproduct of glycolysis, circulates to bone and bone marrow as a nephrogenic factor and leads to the production of FGF23. Further animal studies have shown that exogenous G-3-P may stimulate bone and bone marrow FGF23 production by localized G-3-P acyltransferase-mediated synthesis of lysophosphatidic acid. Together, this study confirmed the presence of a direct kidney–bone signal axis in AKI.

Current studies have suggested that the mechanism of increased circulating FGF23 level in AKI may be the result of the combined effect of increased FGF23 production and reduced renal clearance of circulating FGF23. In addition, inflammatory factors such as IL-6 play an important role in the elevation of FGF23. More recently, researchers have discovered that G-3-P (a kidney-derived metabolite) could circulate to bone and bone marrow then trigger FGF23 production in ischemic AKI. Future studies should continue to investigate specific mechanisms of FGF23 elevation in AKI.

## FGF23 as a therapeutic target in AKI

At present, there is no clinical strategy to reduce the increased FGF23 level in patients with AKI. However, methods used to reduce FGF23 in other diseases may provide us a reference. Among them, neutralizing FGF23 antibody may be a promising therapeutic strategy. However, it may cause hyperphosphatemia especially in patients with non-oliguric AKI who do not need KRT. This has been demonstrated in rats with CKD where the use of FGF23 neutralizing antibody resulted in elevated serum phosphorus followed by a higher risk of aortic calcification and death [57]. Similarly, the administration of a modified anti-FGF23 antibody (burosumab) in patients with X-linked hypophosphatemic rickets, a disease with constantly elevated FGF23 levels, also led to elevated serum phosphate and 1,25(OH)_2_D levels [58]. Therefore, based on available evidence, the only indication for anti-FGF23 antibodies would be AKI with severe oliguria under KRT since hyperphosphatemia could be avoided by in vitro clearance.

Another approach is to block the FGF23 signaling pathway at its receptor binding site or by inhibiting the downstream signaling cascade. A previous study has shown that the C-terminal fragment of FGF23 could compete with full-length FGF23, preventing it from binding to the FGFR–klotho complex; thus, it may be a promising therapeutic agent [59]. Another interfering site in the downstream FGF23 signaling pathway is FGFR4, which may be associated with some off-target effects of FGF23. FGFR4-specific blockers can reduce FGF23-induced left ventricular hypertrophy in 5/6 nephrectomy rat models of CKD [60]. In this way, we could interfere with some off-target (possibly undesirable) effects of FGF23 while maintaining the physiological effects of FGFR1-mediated phosphate and vitamin D homeostasis.

The role of FGF23 antagonists in AKI has not been studied yet. Compared with the extensive study of FGF23 in CKD, the study of FGF23 in AKI is still in its infancy. A critical question arises as to whether the high FGF23 in AKI is just a biomarker, an adaptive compensation, or a maladaptive pathogenic disorder. More in-depth studies are needed to better understand the specific biological and pathophysiological mechanisms of FGF23 in AKI. If increased FGF23 is the pathogenic factor of AKI, we need a selective blocker to inhibit the pathological effect of FGF23 while maintaining its physiological effect.

## Summary

Elevated circulating FGF23 levels are found in both animal models of AKI and patients with AKI. These changes are associated with an increased incidence of AKI, worsening kidney function, and increased mortality. Thus, FGF23 may have potential critical applications in AKI. However, it is too early to include circulating FGF23 measurements in routine AKI measurements. More work is needed to evaluate the clinical value of FGF23 as an early biomarker of AKI or a predictor of adverse outcomes of AKI. Further studies should elucidate the pathophysiological causes and effects of elevated FGF23 in AKI. Only by in-depth understanding of the specific mechanism of FGF23 in AKI can FGF23 be applied to predict the occurrence or prognosis of AKI in clinical settings, or further consider reducing FGF23 as a new target for intervention in AKI.
